# Infectious Complications in Pediatric, Adolescent and Young Adult Patients Undergoing CD19-CAR T Cell Therapy

**DOI:** 10.3389/fonc.2022.845540

**Published:** 2022-03-09

**Authors:** Gabriela M. Maron, Diego R. Hijano, Rebecca Epperly, Yin Su, Li Tang, Randall T. Hayden, Swati Naik, Seth E. Karol, Stephen Gottschalk, Brandon M. Triplett, Aimee C. Talleur

**Affiliations:** ^1^ Department of Infectious Diseases, St. Jude Children’s Research Hospital, Memphis, TN, United States; ^2^ Department of Bone Marrow Transplantation and Cellular Therapy, St. Jude Children’s Research Hospital, Memphis, TN, United States; ^3^ Department of Biostatistics, St. Jude Children’s Research Hospital, Memphis, TN, United States; ^4^ Department of Pathology, St. Jude Children’s Research Hospital, Memphis, TN, United States; ^5^ Department of Oncology, St. Jude Children’s Research Hospital, Memphis, TN, United States

**Keywords:** chimeric antigen receptor (CAR T), infection, immunotherapy, pediatric oncology, B-cell leukemia

## Abstract

CD19-specific chimeric antigen receptor (CAR) T cell therapy has changed the treatment paradigm for pediatric, adolescent and young adult (AYA) patients with relapsed/refractory B-cell acute lymphoblastic leukemia (B-ALL). However, data on the associated infectious disease challenges in this patient population are scarce. Knowledge of infections presenting during treatment, and associated risk factors, is critical for pediatric cellular therapy and infectious disease specialists as we seek to formulate effective anti-infective prophylaxis, infection monitoring schemas, and empiric therapy regimens. In this work we describe our institutional experience in a cohort of 38 pediatric and AYA patients with CD19-positive malignancy treated with lymphodepleting chemotherapy (fludarabine/cyclophosphamide) followed by a single infusion of CD19-CAR T cells (total infusions, n=39), including tisagenlecleucel (n=19; CD19/4-1BB) or on an institutional clinical trial (n=20; CD19/4-1BB; NCT03573700). We demonstrate that infections were common in the 90 days post CAR T cells, with 19 (50%) patients experiencing a total of 35 infections. Most of these (73.7%) occurred early post infusion (day 0 to 28; infection density of 2.36 per 100 patient days-at-risk) compared to late post infusion (day 29 to 90; infection density 0.98 per 100 patient days-at-risk), respectively. Bacterial infections were more frequent early after CAR T cell therapy, with a predominance of bacterial blood stream infections. Viral infections occurred throughout the post infusion period and included primarily systemic reactivations and gastrointestinal pathogens. Fungal infections were rare. Pre-infusion disease burden, intensity of bridging chemotherapy, lymphopenia post lymphodepleting chemotherapy/CAR T cell infusion and development of CAR-associated hemophagocytic lymphohistiocytosis (carHLH) were all significantly associated with either infection density or time to first infection post CAR T cell infusion. A subset of patients (n=6) had subsequent CAR T cell reinfusion and did not appear to have increased risk of infectious complications. Our experience highlights the risk of infections after CD19-CAR T cell therapy, and the need for continued investigation of infectious outcomes as we seek to improve surveillance, prophylaxis and treatment algorithms.

## Introduction

CD19-targeted chimeric antigen receptor (CAR) T cell therapy has provided impressive initial response rates for pediatric and adolescent and young adult (AYA) patients with relapsed/refractory B-cell acute lymphoblastic leukemia (B-ALL) (1–5). However, with growing clinical experience, management and prevention of infectious complications have emerged as key challenges in this population (6). Prior to receiving CD19-CAR T cell therapy, patients have several potential risks factors for infection, including recent intensive therapy, active malignancy, presence of a central venous catheter, and prolonged cytopenias. These are compounded by i) receipt of lymphodepleting chemotherapy prior to CAR T cell infusion, ii) CAR T cell associated inflammation and immune mediated side effects, iii) exposure to immunomodulatory agents to treat CAR T cell-related toxicities (including high-dose corticosteroids and anti-cytokine therapies), and/or iv) anticipated on-target off-tumor effects, such as B cell aplasia (BCA) (7–11).

CD19-CAR T cell therapy studies in pediatric and AYA patients have reported infections in 36-58% of patients, with approximately 20% of patients experiencing grade 3-4 infections (4, 5, 12). In studies of adult patients, severity of cytokine release syndrome (CRS), higher doses of CAR T cells and receipt of multiple prior treatment regimens were associated with increased risk of infections (6, 10, 11, 13). CRS has also been associated with increased infectious risk in pediatric patients in the first month after infusion (6, 14), in part due to receipt of immunosuppressive medications such as corticosteroids and anti-cytokine therapies to treat CAR T cell side effects. Additionally, pediatric patients with a prior history of allogeneic hematopoietic cell transplant (AlloHCT) or immunoglobulin G (IgG) levels <400mg/dL have also been associated with increased risk of infection (14). Immune reconstitution and recovery of bone marrow function have also been identified as important factors in mitigating infections after CAR T cell therapy, though limited information is available in the pediatric setting (8, 15, 16).

Enhancing our understanding of the predictive risk factors, characteristics, timing, and duration of infections in patients receiving CD19-CAR T cells is key in guiding infectious surveillance, treatment, and prophylaxis. Here we report our institutional infectious disease experience in pediatric and AYA patients receiving CD19-CAR T cells. We describe the infectious complications experienced in this cohort and evaluate potential risk factors associated with infection.

## Materials and Methods

This is a retrospective review of patients (n=38) with relapsed and/or refractory CD19-positive malignancy who received lymphodepleting chemotherapy followed by infusion of a CD19-CAR T cell product (tisagenlecleucel or an institutional product [NCT03573700]; total infusions, n=39) at St. Jude Children’s Research Hospital (St. Jude) between October 2018 – August 2021. Additionally, a subset of patients (n=6) received repeat CD19-CAR T cell infusion(s) of the same product due to recurrent malignancy or early loss of BCA. This retrospective project was approved by the St. Jude institutional review board. Written informed consent/assent was obtained from all patients and/or legal guardians to receive treatment with lymphodepletion and CAR T cell therapy, in accordance with institutional guidelines and the Declaration on Helsinki. Both CD19-CAR T cell products utilize a FMC63 svFc and 4-1BB costimulatory domain. Demographic, clinical, laboratory and treatment related data were collected from both a prospective clinical database and retrospective review of the medical record. Data was divided into three time periods: pre-CAR T cells (day -30 to day 0), early post CAR T cells (day 1 to day 28 post infusion) and late post CAR T cells (day 29 to day 90 post infusion).

### Pre-CAR T Cell Infusion Variables (Day -30 to Day 0)

Patient demographics and indications for CAR T cell therapy were recorded. Prior treatment data included receipt of antigen directed therapy (CD19-CAR T cells, blinatumomab, and/or inotuzumab), prior AlloHCT and details of bridging therapy. Bridging therapy was given at the discretion of the treating provider. For this study, bridging therapy was categorized as low or high intensity. Low intensity regimens included no systemic treatment (n=5), focal radiation therapy (n=3) or receipt of systemic chemotherapy agents used during continuation/maintenance therapy for newly diagnosed B-ALL (n=14) (17). High intensity included treatment with agents not included in the low intensity definition. All available absolute neutrophil count (ANC), absolute lymphocyte count (ALC) values and IgG levels were documented. For this study, neutropenia was defined as ANC <500 cells/mm^3^ and lymphopenia as ALC <300 cells/mm^3^. Disease burden included morphologic blast percent from the most recent bone marrow sample, obtained post bridging therapy, when applicable, and prior to CAR T cell treatment.

### CAR T Cell Infusion Related Variables

Lymphodepletion agents and dosages were recorded. All patients received a regimen containing fludarabine (Flu) and cyclophosphamide (Cy). Patients treated on trial all received Flu/Cy (cumulative doses: 75mg/m^2^ and 900mg/m^2^), while patients treated with tisagenlecleucel received Flu/Cy (cumulative doses: 120mg/m^2^ and 1000mg/m^2^, or 75mg/m^2^ and 900mg/m^2^) or Flu/Cy (cumulative doses: 75mg/m^2^ and 900mg/m^2^) with an additional agent (etoposide 500mg/m^2^ or cytarabine cumulative dose, 4000mg). Presence and severity of CAR T cell related immune side effects [CRS, neurotoxicity and CAR-associated hemophagocytic lymphohistiocytosis (carHLH) (18)] were documented, as well as receipt of immunomodulating agents (steroids, tocilizumab, siltuximab, anakinra, and/or ruxolitinib). Available post treatment ANC, ALC and IgG results were collected for up to 90 days post infusion.

### Anti-Infective Prophylaxis and Infection Surveillance Post CAR T Cell Therapy

Our infection prophylaxis approach is shown in **Supplemental Table 1**. Patients received anti-infective prophylaxis for prevention of *Pneumocystis jirovecii* pneumonia (trimethoprim-sulfamethoxazole [TMP-SMX], pentamidine or atovaquone), Herpes simplex virus (HSV) for patients with positive serology or prior history of recurrent episodes of HSV infection (acyclovir or valacyclovir), and fungal disease (echinocandin during lymphodepletion, followed by an azole). Patients receiving antiviral treatment for systemic reactivation prior to CAR T cell therapy remained on the same agent (foscarnet, ganciclovir, or valganciclovir). Antiviral prophylaxis continued until 30 days post CAR T cell infusion. Antifungal prophylaxis continued for at least 30 days post infusion or until evidence of neutrophil recovery (ANC ≥ 500 for 3 consecutive measurements), whichever was longer. Patients did not receive antibacterial prophylaxis post CAR T cell infusion. Patients underwent weekly testing for cytomegalovirus (CMV), Epstein-Barr virus (EBV) and Adenovirus (ADV) by PCR in blood, as well as Aspergillus antigen. All patients had central lines at the time of CAR T cell therapy.

### Definitions of Infections

Infections for which microbiology or histopathology confirmation was available were included in this study. A patient could contribute with one or more infectious episodes. Infection data was collected for the pre- and post-CAR T cell infusion periods.

Blood stream infections (BSI) were defined according to CDC criteria (19). BSIs counted as separate episodes if there was a period of at least 14 days between positive cultures, or if a different organism was identified. Polymicrobial BSI was defined as the detection of different organisms on the first day of a BSI episode. *Clostridiodes difficile* associated diarrhea (CDAD) was included in patients with gastrointestinal symptoms and identification of toxin-producing *C. difficile* by PCR. For infections in other sites, those with compatible symptoms and positive cultures were included.

Systemic viral reactivation was defined as a positive PCR result in blood above the level of detection, irrespective of the presence of symptoms. ADV colitis was reported in patients with gastrointestinal symptoms and a positive quantitative stool PCR. Respiratory viral infections included detection of a virus in a nasopharyngeal sample using the The BioFire^®^ Respiratory 2.1 Panel, in a symptomatic patient. If more than one respiratory virus was detected in the same sample, this was counted as one episode. BK virus infection was reported in patients with hematuria and positive viral PCR in urine or blood.

Patients meeting clinical, laboratory and/or imaging criteria for invasive fungal infection (IFI) according to the European Organization for Research and Treatment of Cancer/Invasive Fungal Infections Cooperative Group and the National Institute of Allergy and Infectious Diseases Mycoses Study Group EORTC/MSG consortium definitions were included (20).

### Management of Infections

Central line and peripheral blood cultures were attempted in all patients with fever, and empiric broad spectrum antibiotic therapy started. Antibiotic therapy was tailored to microbiology results as necessary. If no infectious source was identified, patients received an empiric course of broad-spectrum antibiotic (4^th^ generation cephalosporin) according to institutional guidelines. Systemic viral reactivations were monitored weekly, and therapy started if viral load exceeded institutional established threshold or end-organ disease was suspected. Antifungal prophylaxis was switched to therapy in patients who met clinical criteria for invasive fungal infection.

### Statistical Analysis

The primary aim of our analysis was to describe infectious outcomes experienced by pediatric patients after CD19-CAR T cell infusion, with a focus on initial infusion. Participant data was censored at date of non-response to CAR T cells, development of recurrent detectable disease, the start of post-CAR T cell consolidative AlloHCT preparatory regimen, death or at time of last follow-up. Patients who received more than one infusion during the study period were included and reinfusion data analyzed separately from initial infusion, using descriptive statistics and summary measures.

Basic demographics, clinical information, and laboratory tests (ANC, ALC, IgG) were described using summary statistics, such as median with range and counts with percentage. Cumulative incidence plots were provided to depict the estimated probability of infections of interest in the 90 days after CAR T cell infusion, and number of patients at risk reported by week. Since a patient could die before the occurrence of infection of interest, death was defined as a competing risk. Infection rate was defined as the number of infections divided by the total person-days during the periods of interest, multiplied by 100, and was then calculated for the pre- and post-CAR T cell periods to describe infection density. A Venn diagram was used to illustrate the categories of infections patients had, either detected alone or in combination.

We applied Poisson regression to investigate the effects of pre- and post-CAR T cell therapy risk factors on post infusion infection density. The lab tests of ANC and ALC were first considered as categorial variables (yes/no) using the available data point closest to start of lymphodepletion. We then used all available ANC and ALC data points within the 30 days prior to start of lymphodepletion to define the duration of neutropenia or lymphopenia as a continuous variable. We defined the results in two ways as each captured a different dimension of the information. We considered modeling the response variable of the infections of interest within the early post CAR T cell (1-28 days), and late post CAR T cell (29-90 days) period. All factors of interest were evaluated in univariate analysis, by treating each predictor one at a time and independently. The multivariate model only considered predictors which were statistically significant (*p*-value less than 0.05) in univariate analysis. A forward variable selection method at level of 0.05 was used to determine the final model. In the multivariate model building, ANC and ALC were considered either in categorical form or continuous form, but not both together.

The Fine-Gray competing risk model (21) was utilized to explore what risk factors were associated with the time to the first infection post CAR T cell therapy, with death before the occurrence of infection of interest as a competing risk. Risk factors which occurred before the first infection of interest were evaluated. ANC and ALC test results before the first infection of interest within the early post CAR T cell (1-28 days) and late post CAR T cell (29-90 days) periods were considered similarly in univariate and multivariate analyses, as in the Poisson regression above. Similarly, a multivariate model was fit with predictors which were statistically significant with *(p*-value less than 0.05) in the univariate analysis and a forward variable selection method at 0.05 level was used. To minimize the multicollinearity in the multivariate model, either neutropenia/lymphopenia (Yes or No) or the duration of neutropenia/lymphopenia were considered in separate models with other candidate predictors.

## Results

During the study period, 38 patients with relapsed and/or refractory CD19-positive malignancy received lymphodepleting chemotherapy followed by infusion of CD19-CAR T cells, for a total of 39 initial infusions. CD19-CAR T cell products included tisagenlecleucel (n=19; CD19/4-1BB) or an institutional product (n=20; CD19/4-1BB; NCT03573700). One patient received treatment with both products, with >1 year and receipt of an AlloHCT occurring in between infusions, and therefore contributed twice to initial infusions. Additionally, a subset of patients (n=6) received repeat CD19-CAR T cell infusion(s) of the same product due to recurrent malignancy or early loss of BCA. The clinical outcomes with a focus on disease response to CAR T cell therapy (22, 23), carHLH (18), and epigenetic reprograming of CAR T cells (24), have been reported elsewhere for a subset of these patients.

### Pre-CAR T Cell Therapy

The demographic and clinical characteristics of patients (n=38) receiving initial CD19-CAR T cell infusions (n=39) are summarized in **Table 1**. At time of initial infusion, median age was 9.1 years (range, 1.8 – 23.6). As expected, patients were heavily pretreated, including 10 (25.6%) with prior AlloHCT and 15 (38.5%) having received CD19- and/or CD22-directed therapies. More than half of patients (56.4%) received low intensity bridging chemotherapy, including 5 patients with no systemic therapy and 3 patients that received focal RT. Pre-CAR T cell therapy, patients had a median morphologic leukemic blast percent of 5% (range, 0 – 98) (**Table 1**). Treatment included lymphodepleting chemotherapy followed by a single infusion of CAR T cells. All patients received a fludarabine (Flu) and cyclophosphamide (Cy) based chemotherapy regimen (n=30, Flu/Cy [cumulative doses, 75mg/m^2^ and 900mg/m^2^]; n=7, Flu/Cy [cumulative doses, 120mg/m^2^ and 1000mg/m^2^]; n=2, Flu/Cy [cumulative doses, 75mg/m^2^ and 900mg/m^2^] with etoposide [cumulative dose, 500mg/m^2^] or cytarabine [cumulative dose, 4000mg]).

**Table 1:** Demographics and treatment characteristics.

Demographics, N=38 patients	
Sex	
Female	18 (47.4)
Male	20 (52.6)
Race	
White	30 (78.9)
Black	5 (13.2)
Other (Asian, American Indian/Alaskan Native or Multiple Race)	3 (7.9)
Ethnicity	
Hispanic (Mexican/Chicano, Puerto Rican, South/Central American)	13 (34.2)
Non-Hispanic	25 (65.8)
Primary Diagnosis	
B-ALL	37 (97.4)
B-Lymphoblastic Lymphoma	1 (2.6)
**Treatment Characteristics,** N=39 initial infusion episodes	
Age at Infusion (median [range])	9.06 years [1.8 – 23.6]
Indication for CART	
Primary refractory	5 (12.8)
Relapsed disease	34 (87.2)
Relapse 1	17 (43.6)
Relapse ≥ 2	17 (43.6)
Pre-CART morphologic blast % (median [range])	5 [0 – 98]
Prior Therapy	
Allogeneic HCT	10 (25.6)
Antigen Directed*	15 (38.5)
Blinatumomab	12 (30.8)
Inotuzumab	5 (12.8)
CD19-CART	1 (2.5)
Bridging Chemotherapy	
High intensity	17 (43.6)
Low intensity	22 (56.4)
CRS Max Grade	
0	14 (35.9)
1-2	19 (48.7)
3-4	6 (15.4)
Neurotoxicity Max Grade	
0	30 (76.9)
1-2	5 (12.8)
3-4	4 (10.3)
carHLH	5 (12.8)
Post-CART Immunomodulatory Treatments	
Tocilizumab	13 (33.3)
Corticosteroids	5 (12.8)
Siltuximab	4 (10.2)
Anakinra	5 (12.8)
Ruxolitinib	1 (2.6)

Numerical data are presented as the n (%) unless otherwise specified. B-ALL, B-cell acute lymphoblastic leukemia; CART, chimeric antigen receptor T-cell therapy; HCT, hematopoietic cell transplantation; CRS, cytokine release syndrome; carHLH, CAR associated hemophagocytic lymphohistiocytosis; ^morphologic blast % from most recent marrow prior to start of lymphodepleting chemotherapy; *patients may have received more than one antigen directed therapy.

Immediately prior to the start of lymphodepleting chemotherapy, 33.3% of patients (n=13) were neutropenic (ANC <500 cells/mm^3^) and 20.5% (n=8) were lymphopenic (ALC <300 cells/mm^3^). When accounting for all available results within 30 days prior to start of lymphodepleting chemotherapy, 20 patients had neutropenia and lymphopenia, with a median duration of 13.5 (range, 1 – 30) and 7 (range, 1 – 30) days, respectively. Additionally, a subset of patients (n=26) had available pre-CAR T cell IgG levels, of which 23.1% were low (IgG <400 mg/dL). As expected, after CAR T cell infusion most patients experienced neutropenia and lymphopenia (**Table 2**), with a median duration of 14 (range, 1 – 69) and 11 (range, 2 – 53) days, respectively.

**Table 2:** Hematologic parameters pre- and post-CAR T cell therapy.

	ANC <500 cells/mm^3^	ALC <300 cells/mm^3^	IgG <400 mg/dL
**Pre-CART Therapy** (n=39)^#^			
Pre-lymphodepletion^*^, n (%)	13 (33.3)	8 (20.5)	6 (23.1)
Duration^^^, median (range);	13.5 (1 – 30)	7 (1 – 30)	–
**Post-CART Therapy**			
Time period, n (%)			
Days 0 – 7 (n=39)	28 (71.8)	39 (100)	5 (14.7)
Days 8 – 21 (n=39; IgG, n=32)	32 (82)	24 (61.5)	3 (8.8)
Days 22 – 63 (n=37; IgG, n=34)	15 (40.5)	14 (37.8)	16 (47.1)

#for ANC/ALC, n=39; for IgG, n=26; *Pre-lymphodepletion, last available result prior to CART associated lymphodepleting chemotherapy; ^duration, in those with neutropenia or lymphopenia (n=20) at any time in the 30 days prior to CART infusion, the sum of days between the first value meeting defined low criteria and the first value above that criteria; CART, chimeric antigen receptor T-cell; ANC, absolute neutrophil count; ALC, absolute lymphocyte count; IgG, Immunoglobulin G.

In the 30 days prior to CAR T cell infusion, 12 (30.8%) patients had a total of 20 infectious episodes. Most infections identified in this period were viral (n=10, 17 infectious episodes). Seven patients (41%) had systemic viral reactivations, 5 of which had a prior AlloHCT and previous history of viral reactivation. No end organ disease associated with viral reactivation was detected, and 3 received antiviral therapy while undergoing CAR T cell therapy. Only 7.7% of patients (n=3) had a bacterial infection, 2 BSIs (*A. xylosoxidans* and *S. epidermidis*) and 1 C. difficile-associated diarrhea (CDAD) (**Table 3**).

**Table 3:** Infections pre- and post-CAR T cell therapy.

Type of Infection	Days -30 - 0 Pre-CART (N=39)		Days 1-28 Post CART (N=39)		Days 29-90 Post CART (N=33)	
	Total Episodes	Patients Affected	Total Episodes	Patients Affected	Total Episodes	Patients Affected
**Any Infection**	20	12 (30.7)	23	14 (35.9)	12	8 (24.3)
**Bacterial Infections**	3	3 (7.7)	14	9 (23.1)	5^+^	4 (12.2)
Bacteremia	2	2 (5.1)	9	7 (17.9)	3	3 (9.1)
Other*	1	1 (2.5)	5	3 (7.7)	3	3 (9.1)
**Viral Infections**	17	10 (25.6)	8	7 (17.9)	6	6 (18.2)
Systemic	11	7 (17.9)	4	4 (10.3)	1	1 (3)
Respiratory	3	3 (7.7)	1	1 (2.6)	1	1 (3)
Other*	3	3 (7.7)	3	3 (7.7)	4	4 (12.2)
**Fungal Infections**	0	0	1	1 (2.6)	1	1 (3)

Numerical data are presented as the n (%). *Other infections include the following sites: skin and soft tissue, gastrointestinal, central nervous system, sinuses; +One patient had MSSA skin and bloodstream infections concomitantly.

### Infections Post CAR T Cell Therapy

**Figure 1:**
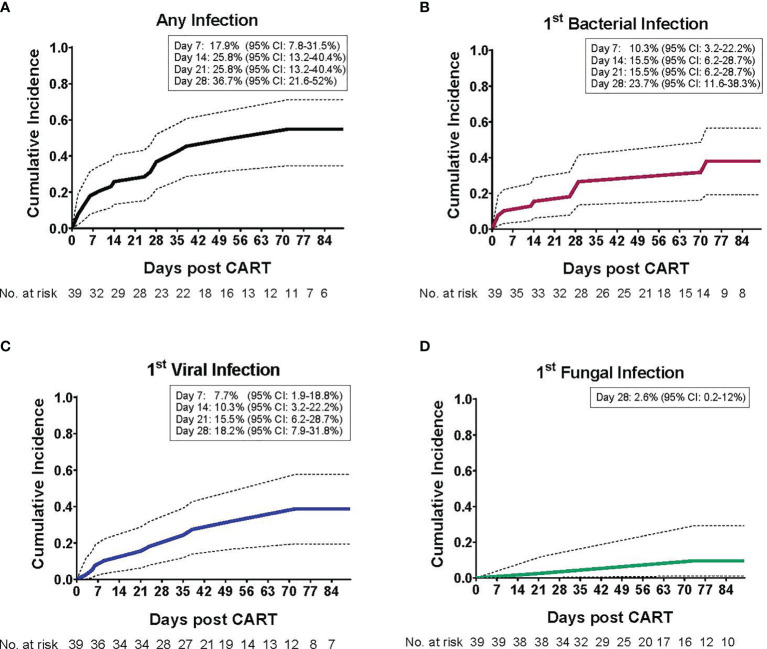
Cumulative incidence of infections post CD19-CAR T cell therapy in pediatric and AYA patients. The cumulative incidence (CI) of infection post CD19-CAR T cell therapy, as a function of the day of infection onset, is depicted for the entire study period (day 1 – 90). **(A)** The cumulative incidence of first infection, such that each patient may contribute only once (either bacterial, viral and/or fungal infection, whichever occurred first). Most patients (73.7%) experienced their first infection within the initial 28 days post CAR T cells, with a 36.7% CI (95% confidence interval: 21.6 - 52). The cumulative incidence of first **(B)** bacterial infection, with a 23.7% CI (95% confidence interval: 11.6 – 38.3), **(C)** viral infection, with a 18.2% CI (95% confidence interval: 7.9 – 31.8), and **(D)** fungal infection, with a 2.6% CI (95% confidence interval: 0.2 – 12), by day 28 post CAR T cell infusion.

**Figure 2:**
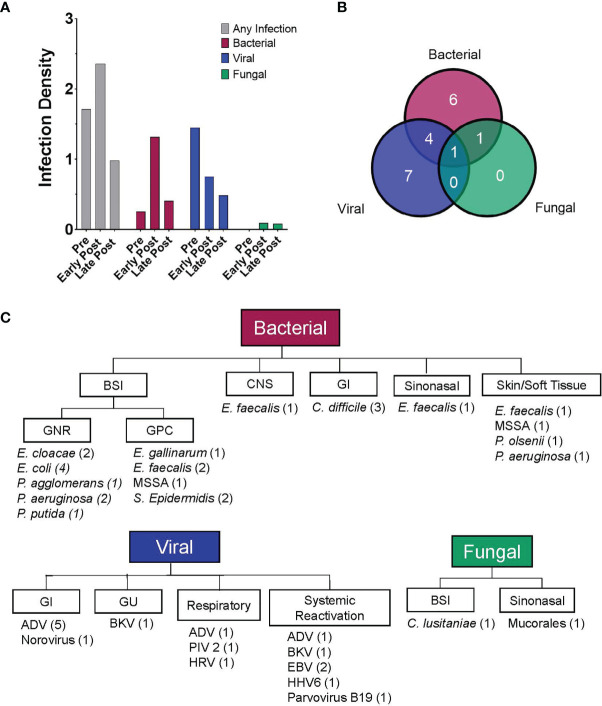
Patients experienced a high rate of infections post CD19-CAR T cell therapy. **(A)** Bar graphs depicting the infection density per 100 days-at-risk, for any infection and by pathogen category (bacterial, viral and fungal). Data is displayed for three time periods, pre-CAR T cells (day -30 to day 0), early post CAR T cells (day 1 to day 28) and late post CAR T cells (day 29 to day 90). Infections occurred in 30.8% (infection density, 1.709), 34.2% (infection density, 2.358), and 24.2% (infection density, 0.978) of infusions, in the pre-, early post and late post CAR T cell therapy periods, respectively. **(B)** Venn diagram showing the number of patients experiencing at least one infection over the entire study period (n = 19) and depicting the types of infection experienced by each patient (bacterial, viral and/or fungal). **(C)** Flow diagram displaying details of infections experienced by pediatric and AYA patients after CD19-CAR T cell therapy (day 0 to day 90 post infusion). The figure depicts infection category, sites of infection, infectious pathogens (number of patients identified with that pathogen), for all patients after CD19-CAR T cell therapy.

The cumulative incidence of first infection, overall and by infection type, in the 90 days post CAR T cells are shown in **Figure 1**. The cumulative incidence of first infection was 17.9% (95% CI: 7.8-31.5%) by day 7, 25.8% (95% CI: 13.2-40.4%) by days 14 and 21, and 36.7% (95% CI: 21.6-52%) by day 28 post CAR T cell therapy. Infections were primarily bacterial (**Figure 1B**) followed by viral (**Figure 1C**), with a very low incidence of fungal infection (**Figure 1D**). Infection density in the 30 days prior to CAR T cell infusion was 1.71 per 100 days-at-risk. After CAR T cell therapy, most infections occurred early (infection density of 2.36 per 100 patient days-at-risk) compared to late post infusion (infection density 0.98 per 100 patient days-at-risk) (**Figure 2A**). The majority of patients had more than one type of infection (**Figure 2B**). Details of bacterial and viral infections are provided in **Supplemental Tables 2, 3**.

In the early post CAR T cell therapy period (day 1 – 28), 14 patients had a total of 23 infectious episodes (**Table 3**). Most infections were bacterial, with 9 patients (23.1%) contributing a total of 14 infectious episodes. This includes 9 episodes of bacteremia, 3 with gram positive and 6 with gram negative bacteria (**Figure 2C**). Most BSI episodes (78%) occurred in the setting of concurrent neutropenia. Notably, only 2 of the 9 organisms were susceptible to levofloxacin and of these, one was isolated in a non-neutropenic patient (**Supplemental Table 2**). Seven patients (17.9%) had a viral infection (n=8), most due to viral reactivation (**Supplemental Table 3**). One patient developed invasive rhinocerebral mucormycosis, identified by histopathology (**Figure 2C**). Notably, this patient had extended neutropenia pre-CAR T cell therapy, received immunomodulatory therapy for treatment of CAR-related side effects and only intermittently received fungal prophylaxis with an azole due to oral intolerance. No other fungal infections were found during this period (**Table 3**).

Thirty-three patients contributed data to the late post CAR T cell therapy period (day 29 – 90), with 8 patients (24.2%) experiencing a total of 12 infectious episodes. Notably, the number at risk in this period declined over time primarily due to lack of response to CAR T cell infusion or receipt of a consolidative AlloHCT. Of the 12 documented infections, 6 were viral and 5 were bacterial (3 BSIs). One episode of candidemia was identified late post CAR T cells (**Table 3**).

### Post CAR T Cell Immune Mediated Side Effects and Infections

**Figure 3:**
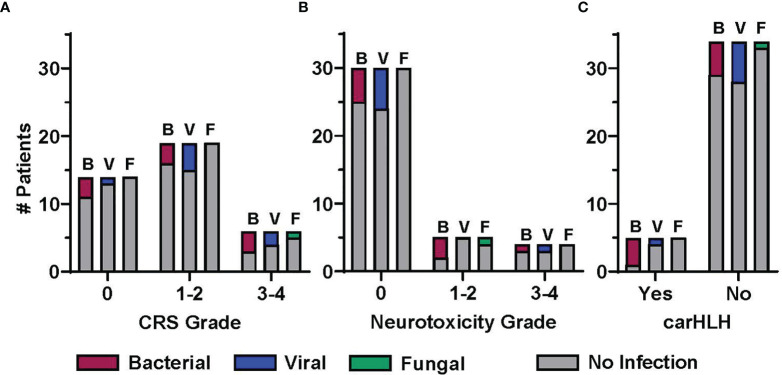
Association between infection and post CD19-CAR T cell therapy immune mediated side effects. The proportion of patients experiencing at least one infection in the early post CAR T cell therapy period (day 1 to day 28), displayed by infection category and severity of CAR T cell therapy related immune mediated side effects (CRS, cytokine release syndrome; NTX, neurotoxicity; carHLH, CAR-associated hemophagocytic lymphohistiocytosis). Patients with at least 1 infection in a given category (B, bacterial; V, viral; F, fungal) are presented in the colored bars, with a patient contributing up to one infection per category. Patients not having an infection in the given category are accounted for in the gray bar. Distribution is by patients by highest grade **(A)** CRS and **(B)** neurotoxicity, or presence/absence of **(C)** carHLH.

CAR T cell immune mediated side effects included CRS in 64% (n=25) of patients, neurotoxicity in 23% (n=9) and carHLH in 12.8% (n=5). Most cases were low grade, with only 15.4% of patients experiencing grade ≥3 CRS and 10.3% grade ≥3 neurotoxicity. Thirteen patients received immunomodulatory therapy, including tocilizumab (n=13), corticosteroids (n=5), siltuximab (n=4), anakinra (n=5), and/or ruxolitinib (n=1) (**Table 1**). The proportion of patients with and without infection early post CAR T cell infusion, stratified by presence and/or grade of CRS, neurotoxicity and carHLH, is shown in **Figure 3**. Eleven (44%) patients with CRS experienced at least one infectious episode. This includes 5, 6, and 1 patient(s) with at least one bacterial, viral, and fungal infection, respectively. A greater portion of patients were affected with increasing max CRS grade, with 7 of 19 (37%) patients with grade 1-2 CRS having infections, compared to 4 of 6 (67%) patients with grade 3-4 CRS (**Figure 3A**). Five (55.5%) patients with neurotoxicity and 4 (80%) patients with carHLH had at least one documented infection post CAR T cell infusion. Most infections in patients with neurotoxicity (4 of 5 patients; **Figure 3B**) and carHLH (3 of 4 patients; **Figure 3C**) were bacterial.

### Outcomes Related to Infections Post CAR T Cell Therapy

Seven (17.9%) patients were admitted to the intensive care unit (ICU) after CAR T cell infusion. Three of these patients had active infections at time of ICU admission, and 2 died from infectious complications. This included 1 patient with extensive rhinocerebral mucormycosis and concomitant *E. faecalis* disseminated infection (BSI, CNS and sinonasal), and 1 patient with polymicrobial BSI with *E. faecalis* and *S. epidermidis* in the days prior to death. One additional patient had no documented infection at time of death, but postmortem examination revealed *E. faecalis* in cultures. All 3 patients that died with infection also had immune-mediated complications (CRS [n=3], neurotoxicity [n=2], carHLH [n=2]) treated with immunomodulating therapy and, in the 2 weeks prior to the last identified infection, had received a course of broad-spectrum antibiotics for documented infection or as empiric management of fever and neutropenia.

### Risk Factors for Infections Post CAR T Cell Therapy

We sought to evaluate possible associations between pre- and post-CAR T cell therapy variables and infection density, within the early and late post therapy time periods. In univariate analysis, numerous variables, including duration of neutropenia pre- and post-CAR T cell therapy, intensity of bridging chemotherapy, immune mediated side effects and immunomodulatory treatment, were associated with increased infection density in the early post CAR T cell period. However, in the multivariate model, only pre-therapy disease burden (rate ratio: 1.02 [95% CI: 1.01, 1.03]; *p <*0.01) remained statistically significant. Conversely, in the late post CAR T cell therapy period, fewer evaluated variables were found to be associated with increased infection density in univariate analysis. However, pre-therapy lymphopenia (9.3 [2.34, 36.94]; *p <*0.01), carHLH (37.36 [3.56, 391.83]; *p <*0.01) and duration of low IgG (1.05 [1.02, 1.09]; *p <*0.01) were statistically significant in the multivariate model (**Table 4**).

**Table 4:** Association of pre- and post-CAR T cell therapy variables with post CAR T cell therapy infection density.

Variables^	Early Post CART (day 1 to 28)		Late Post CART (day 29 to90)	
	Univariate Ratio (95% CI); *P*-value	Adjusted Ratio (95% CI)*; *P*-value	Univariate Ratio (95% CI); *P*-value	Adjusted Ratio (95% CI)*; *P*-value
**Pre-CART variables**				
ANC<500 cells/mm^3^	3 (1.3, 6.93); **0.0101**		2.23 (0.71, 7.03); 0.1702	
ALC<300 cells/mm^3^	0.67 (0.2, 2.24); 0.5105		5.29 (1.68, 16.66); **0.0045**	9.3 (2.34, 36.94); **0.0015**
IgG<400 mg/dL	0.22 (0.03, 1.69); 0.1471		0.44 (0.06, 3.45); 0.4354	
Duration of ANC<500 cells/mm^3^	1.06 (1.02, 1.09); **0.0014**		–	
Duration of ALC<300 cells/mm^3^	1.04 (1.01, 1.08); **0.022**		–	
Duration of IgG<400 mg/dL	0.88 (0.71, 1.09); 0.241		–	
Infection Pre-CART	0.96 (0.39, 2.33); 0.927		1.27 (0.38, 4.23); 0.6926	
Prior AlloHCT	0.49 (0.14, 1.64); 0.2439		2.66 (0.84, 8.38); 0.095	
Bridging chemotherapy (High vs. Low intensity)	3.92 (1.54, 9.93); **0.004**		1.42 (0.45, 4.46); 0.5523	
Pre-CART marrow blast %	1.02 (1.01, 1.03); **<0.0001**	1.02 (1.01, 1.03); **<0.0001**	1 (0.98, 1.03); 0.8288	
**Post-CART variables**				
CRS	2.78 (0.94, 8.16); **0.0634**		3.31 (0.73, 15.11); 0.1222	
Neurotoxicity	2.73 (1.2, 6.23); **0.0169**		–	
carHLH	3.02 (1.19, 7.65); **0.02**		9.21 (1.19, 71.33); **0.0335**	37.36 (3.56, 391.83); **0.0025**
Tocilizumab	3.37 (1.46, 7.78); **0.0045**		1.02 (0.28, 3.78); 0.9721	
Corticosteroids	4.68 (2.03, 10.81); **0.0003**		–	
Siltuximab	4.55 (1.87, 11.06); **0.0008**		–	
Anakinra	3.02 (1.19, 7.65); **0.02**		9.21 (1.19, 71.33); **0.0335**	
Duration^#^ of ANC<500 cells/mm^3^	1.06 (1.02, 1.1); **0.004**		0.99 (0.95, 1.03); 0.6406	
Duration^#^ of ALC<300 cells/mm^3^	1 (0.94, 1.07); 0.9315		1.01 (0.98, 1.04); 0.6485	
Duration^#^ of IgG<400 mg/dL	1 (0.87, 1.15); 0.9984		1.03 (1, 1.06); **0.0241**	1.05 (1.02, 1.09); **0.0033**

Poisson Regression. ^variables categorized as Yes vs No, unless otherwise specified; #duration in the specified time period; *Multivariate analysis includes variables with p > 0.05 in univariate analysis. CART, chimeric antigen receptor T-cell therapy; ANC, absolute neutrophil count; ALC, absolute lymphocyte count; IgG, immunoglobulin G; AlloHCT, allogeneic hematopoietic cell transplant; CRS, cytokine release syndrome; carHLH, CAR associated hemophagocytic lymphohistiocytosis.

Bold = statistically significant.

Additionally, we investigated the impact of pre- and post-CAR T cell variables on time to first infection. In the early post CAR T cell therapy period, high intensity bridging chemotherapy (rate ratio: 4.78 [95% CI: 1.41, 16.19]; *p* = 0.012) and duration of post CAR T lymphopenia (0.81 [0.7, 0.95]; *p* = 0.011) were associated with increased infection risk (**Table 5**). In the late post CAR T cell therapy period, no variables were statistically associated with time to first infection.

**Table 5:** Association of pre- and post-CAR T cell therapy variables with time to first infection post CAR T cell therapy.

Variables^	Early Post CART (day 1 to 28)		Late Post CART (day 29 to 90)	
	Univariate Hazard Ratio (95% CI); *P*-value	Adjusted Hazard Ratio (95% CI)*; *P*-value	Univariate Hazard Ratio (95% CI); *P*-value	Univariate Hazard Ratio (95% CI); *P*-value
**Pre-CART variables**				
ANC<500 cells/mm^3^	1.73 (0.61, 4.89); 0.3025		2.32 (0.6, 9.01); 0.2236	
ALC<300 cells/mm^3^	0.99 (0.32, 3.11); 0.9911		2.83 (0.83, 9.7); 0.0971	
IgG<400 mg/dL	0.37 (0.05, 2.69); 0.3248		0.78 (0.1, 6.18); 0.8166	
Duration of ANC<500 cells/mm^3^	1.03 (0.99, 1.08); 0.1101		1.03 (0.98, 1.09); 0.2735	
Duration of ALC<300 cells/mm^3^	1.02 (0.98, 1.08); 0.3278		1.03 (0.95, 1.11); 0.46	
Duration of IgG<400 mg/dL	0.92 (0.79, 1.06); 0.2547		1.01 (0.89, 1.14); 0.9361	
Infection Pre-CART	0.98 (0.3, 3.21); 0.9781		1.56 (0.4, 6.08); 0.5221	
Prior AlloHCT	0.44 (0.1, 2.01); 0.2912		0.9 (0.22, 3.64); 0.8777	
Bridging chemotherapy (High vs. Low intensity)	4.16 (1.26, 13.71); **0.0191**	4.78 (1.41, 16.19); **0.0119**	3.42 (0.89, 13.11); 0.0723	
Pre-CART marrow blast %	1.02 (1.01, 1.03); **0.0012**		1 (0.98, 1.03); 0.8571	
**Post-CART variables**				
CRS	1.41 (0.49, 4.07); 0.5250		4.66 (0.57, 38.15); 0.1515	
Neurotoxicity	1.76 (0.59, 5.24); 0.3064		–	
carHLH	3.24 (1.33, 7.94); **0.0099**		3.17 (0.36, 27.69); 0.2969	
Tocilizumab	1.5 (0.53, 4.29); 0.4463		1.38 (0.35, 5.38); 0.642	
Corticosteroids	2.56 (0.94, 6.97); 0.0654		–	
Siltuximab	1.08 (0.17, 7.08); 0.9347		–	
Anakinra	3.24 (1.33, 7.94); **0.0099**		3.17 (0.36, 27.69); 0.2969	
Duration^#^ of ANC<500 cells/mm^3^	0.95 (0.89, 1.02); 0.1448		0.83 (0.63, 1.08); 0.1612	
Duration^#^ of ALC<300 cells/mm^3^	0.83 (0.69, 0.99); **0.0373**		0.97 (0.91, 1.03); 0.2639	
Duration^#^ of IgG<400 mg/dL	0.71 (0.46, 1.09); 0.1203		0.97 (0.83, 1.14); 0.7319	

Cox proportional hazards model. ^variables categorized as Yes vs No, unless otherwise specified; #duration in the specified time period; *Multivariate analysis includes variables with p > 0.05 in univariate analysis. CART, chimeric antigen receptor T-cell therapy; ANC, absolute neutrophil count; ALC, absolute lymphocyte count; IgG, immunoglobulin G; AlloHCT, allogeneic hematopoietic cell transplant; CRS, cytokine release syndrome; carHLH, CAR associated hemophagocytic lymphohistiocytosis.

Bold = statistically significant.

### Infections Post Repeat CAR T Cell Infusions

After initial infusion, 6 patients received subsequent treatment with lymphodepleting chemotherapy followed by CAR T cell reinfusion. One patient contributed data for 3 reinfusions, for a total of 8 reinfusions among the 6 patients. Four patients developed infection following CAR T cell reinfusion, for a total of 12 infections. Interestingly, no patient had infection in the early post CAR T cell time period. Bacterial infections included 3 patients with a BSI (1 *Pseudomonas mendocina*, 1 Viridians Group Streptococcus [VGS], and 1 polymicrobial: VGS and *K. pneumonia*) and 3 with CDAD. Three patients had viral infections, including 2 patients with systemic reactivations and 1 with viral respiratory pathogens. No patients in this cohort had a fungal infection (**Supplemental Table 4**).

## Discussion

In this single institution retrospective analysis, we report on the infectious complications of 38 pediatric and AYA patients with relapsed/refractory CD19-positive malignancy, who received lymphodepleting chemotherapy followed by CD19-CAR T cell infusion. The incidence of infections post CAR T cell therapy in this cohort is similar to previous reports, with the highest number of infections occurring early post infusion (4–6, 25). Bacterial infections were the most frequent overall, typically occurring within 28 days of CAR T cell therapy and with primarily BSIs. Viral infections occurred at similar rates across the study period and included mainly systemic viral reactivations and gastrointestinal pathogens. Fungal infections were rare. Despite the small sample size and heterogenous nature of our patient population, our findings are consistent with the reported experience of other pediatric centers, demonstrating that the overall proportion and etiology of infections, as well as attributable mortality, is similar despite treatment at different centers. Given the similarities with previously published data, this work further establishes the expected infectious trends in the pediatric and AYA population, which can be used by practitioners to inform upon clinical care and decisions.

In accordance with others, most bacterial infections in our patient population presented as BSI, with organisms similar to those reported in patients receiving chemotherapy (6, 14, 25, 26). However, viral infections in our cohort were mainly due to systemic reactivations and gastrointestinal viruses, mainly ADV, with very few respiratory viruses (6, 14). This may be in part due to our routine, prospective monitoring for viral reactivations and highlights the need for such strategies. As reported by others, reactivation of double stranded DNA virus did not lead to end organ disease (6). While HSV and VZV have been described in children and adults after CAR T cell infusions to therapy, we did not observe any cases, likely due to the use of acyclovir prophylaxis (14). Given our data is limited to 90 days post infusion, this may influence outcomes related to viral infections. Notably, several patients had known systemic viral reactivation in the 30 days prior to CAR T cell therapy, some of which were detected as part of screening tests during pre-CAR T cell therapy evaluation. A minority of these patients required antiviral therapy, but all were monitored weekly to determine the need for preemptive therapy. Invasive fungal infection, albeit rare, carried significant morbidity and mortality, consistent with prior reports (6, 27, 28). The patient with invasive fungal infection was significantly immunosuppressed pre-therapy, and post infusion had persistent neutropenia and higher-grade CRS/neurotoxicity requiring immunomodulatory treatments, further supporting these variables as risks factor for severe infection (4, 6, 10, 11, 14). Importantly, among those patients that received repeat CD19-CAR T cell infusions, incidence of infection did not appear higher than in those with initial infusion. Larger patient cohorts are needed to evaluate this further.

We identified several factors associated with increased risk of higher infection density in the early post CAR T cell therapy period. However, in multivariate analysis only pre-CAR T cell therapy disease burden remained significant. Given the size and heterogeneity of our cohort, associated variables such as pre-CAR T cell therapy neutropenia and intensity of bridging chemotherapy may not have maintained significance, despite representing a similar patient profile to those with higher leukemia burden. Notably, receipt of high intensity bridging chemotherapy impacted time to development of first infection. Increased anti-malignancy therapies pre-CAR T cell therapy has also been reported by others as an independent risk factor for infection post CAR T cell infusions (25). In contrast to previous reports, prior AlloHCT, recent history of infections and pre-existing neutropenia were not associated with increased risk of infection (6, 14). These observations are limited due to the number and heterogeneity of patients included in this study. However, as our study includes all patients who received CD19-CAR T cell therapy at our institution, inclusive of 2 products, with a similar approach to antimicrobial prophylaxis and extended follow up to 90 days, we believe that our findings are still meaningful.

While CRS has been described as a risk factor for infection (6, 13), in our study immune mediated side effects and associated immunomodulatory treatments did not maintain statistical significance in multivariate analysis in the early post CAR T cell period. However, our limited number of patients with high grade CRS/neurotoxicity and therefore minimal use of corticosteroids may have favorably impacted infection risk. Furthermore, patients with fever received an empiric antibiotic course which may have mitigated the risk of developing infection during this high-risk period. It is therefore still prudent to have a high index of suspicion for infection in this population. Importantly, patients with carHLH did have a higher number of infections in the late post CAR T cell therapy period. This is likely since carHLH often occurs later than CRS and may require treatment with immunosuppressive agents such as anakinra and steroids (18, 29), as we saw in our patient population. The use of immunomodulatory agents to treat CAR-mediated side effects, the inflammatory response with elevated cytokines and/or the intensive supportive management in the ICU may all contribute to risk of infections in these patients (30–32). As we use such agents earlier in the course of treatment, including as prophylaxis, it will be important to continue to evaluate for impact on infectious outcomes particularly in larger patient cohorts (33–35).

Recognizing the substantial risk for infectious complication after CAR T cell therapy, we and others have developed institutional guidelines for prophylactic and empiric treatment regimens. The use of antibacterial prophylaxis in the setting of CAR T cell therapy remains controversial (7–9). While standard practice at some institutions, a recent report highlights that use of antibacterial prophylaxis may not significantly decrease rates of bacterial infection post CAR T cell therapy (25). Our data support this, as most bacterial infections in our patient cohort would not have been prevented using levofloxacin prophylaxis. Of particular importance is the use of a mold-active anti-fungal agent as soon as feasible after CAR T cell infusion, especially among those patients deemed to be at high-risk for invasive fungal infections. We also recognize that routine use and choice of prophylactic agents may impact timing and nature of post CAR T cell infectious complications. Furthermore, with the advent of new CAR T cell products and treatment of varied patient populations and disease indications, it will be important to reassess clinical management guidelines to maintain best clinical practice among different patient groups.

BCA is an expected side effect after CD19-CAR T cell therapy (3, 4, 36, 37) and recognized as a risk factor for infection (6, 8, 14). Our analysis of the possible relationship between infection and hypogammaglobulinemia is limited by the fact that, among those patients that had disease response to CAR T cell therapy, 15 proceeded to consolidative AlloHCT. Therefore, we have inadequate extended data to address this question. The role of prolonged hypogammaglobulinemia in the risk of infections beyond 90 days needs to be further addressed in larger cohorts with longer follow up periods. Specifically, several questions remain regarding the role of infections due to encapsulated bacteria, many of them immune preventable, and how BCA increases the risk and impacts revaccination in these patients. Currently, there is no evidence to guide immunization in this setting. Expert opinion, derived from knowledge on immunization after AlloHCT, established recommendations for revaccination of these patients (7, 38). However, the safety and immunogenicity of vaccines in patients undergoing CAR T cell therapy is largely unknown (7, 11, 39, 40). Prospective studies are needed to address these questions.

In conclusion, we describe the incidence and distribution of infectious complications in pediatric and AYA patients receiving CD19-CAR T cell therapy at our institution. Infections were seen throughout the first 90 days post CAR T cell infusion, with bacterial infections being most common and occurring primarily in the early post CAR T cell therapy period. Pre-therapy disease burden, intensity of bridging chemotherapy, post CAR T cell therapy lymphopenia and development of carHLH were all significantly associated with either infection density or time to first infection. Our study adds to the growing literature and aids in defining patients at higher risk for infections after CD19-CAR T cell therapy, which is critical to the establishment of adequate protocols for infection surveillance, prophylaxis, and treatment.

## Data Availability Statement

The raw data supporting the conclusions of this article will be made available by the authors, without undue reservation.

## Ethics Statement

The studies involving human participants were reviewed and approved by Institutional Review Board (IRB), St Jude Children’s Research Hospital. Written informed consent from the participants’ legal guardian/next of kin was not required to participate in this retrospective study in accordance with the national legislation and the institutional requirements.

## Author Contributions

GM, DH, RE, SN, SK, RH, SG, BT, and AT provided clinical care of patients. GM and AT performed data collection. YS and LT performed statistical analysis. GM, DH, RE, YS, LT, and AT analyzed and interpreted data. GM, DH, RE, and AT wrote the manuscript. All authors contributed to the article and approved the submitted version.

## Funding

This work was supported by the National Institutes of Health (NIH)/National Cancer Institute grants P30CA021765 and 5P30CA021765-42, the American Society of Transplantation and Cellular Therapy (AT), the American Society of Hematology (AT), and the American Lebanese Syrian Associated Charities. The content is solely the responsibility of the authors and does not necessarily represent the official views of the NIH.

## Conflict of Interest

SG consults/consulted for TESSA Therapeutics, TIDAL, Catamaran, and Novartis and is DSMB member of Immatics. SG and RE have patents/patent applications in the fields of T-cell and/or gene therapy for cancer. GM receives research funding from Astellas Inc and SymBio Pharmaceuticals Limited. DH receives research funding from Merck, AstraZeneca and Regeneron. RH serves on advisory boards for Roche Diagnostics, Quidel, Diagnostics, and Inflammatix.

The remaining authors declare that the research was conducted in the absence of any commercial or financial relationships that could be construed as a potential conflict of interest.

## Publisher’s Note

All claims expressed in this article are solely those of the authors and do not necessarily represent those of their affiliated organizations, or those of the publisher, the editors and the reviewers. Any product that may be evaluated in this article, or claim that may be made by its manufacturer, is not guaranteed or endorsed by the publisher.
