# Supplementary material for: Infectious Complications in Pediatric, Adolescent and Young Adult Patients Undergoing CD19-CAR T Cell Therapy
Source: Front Oncol. 2022 Mar 9;12:845540. doi: 10.3389/fonc.2022.845540 (PMC8959860; doi:10.3389/fonc.2022.845540)
Supplement: Supplementary file 1 [file Table_1.docx]

**Supplemental Table 1. Infection prophylaxis for CD19-CAR T cell patients at St. Jude Children’s Research Hospital**

| **Infection Category** | **Recommended agent^#^** | **Duration** |
| --- | --- | --- |
| Antiviral prophylaxis* | Acyclovir (oral) | Commence with start of lymphodepleting chemotherapy and continue for at least 30 days post CAR T cell infusion |
| Anti-*Pneumocystis* prophylaxis^^^ | Trimethoprim/Sulfamethoxazole (oral; 3 times/week)  Or, if allergic or intolerant:  Pentamidine (intravenous; monthly) | Commence with start of lymphodepleting chemotherapy and continue until CD4 > 200 cells/µL |
| Antifungal prophylaxis | Micafungin (intravenous),  followed by  Voriconazole (oral) | Commence micafungin with start of lymphodepletion, then switch to voriconazole^±^ as soon as possible  Continue until neutrophil recovery (ANC ≥ 500 cells/µL for at least 3 consecutive measurements) |

CAR, chimeric antigen receptor; ANC absolute neutrophil count. *If patient was receiving other antiviral medications (ganciclovir, valganciclovir, foscarnet) as prophylaxis due to prior viral reactivation, those were continued through the course of CAR T-cell therapy. ^For patients unable to take Trimethoprim/Sulfamethoxazole or pentamidine, atovaquone is an alternative. ±Posaconazole was used on cases already receiving the drug or intolerance to voriconazole. #If patient unable to tolerate oral medication, consider conversion to an intravenous formulation.

**Supplemental Table 2. Blood Stream Infections in Days 1-90 post CAR T cell Therapy**

| **Organism(s)**+ | **Days post**  **CART** | **ANC^** | **ICU admission for infection** | **CRS**  **(max grade)** | **NTX**  **(max grade)** | **carHLH** |
| --- | --- | --- | --- | --- | --- | --- |
| Days 1-28 | | | | | | |
| *Staphylococcus epidermidis* | 13 | 0 | No | 4 | 4 | Yes |
| *Staphylococcus epidermidis*  *Enterococcus faecalis* | 14 | 10 | Yes | 1 | 0 | Yes |
| *Pseudomonas aeruginosa*  *Enterococcus faecalis* | 2  15 | 0  10 | Yes  NA | 3 | 2 | No |
| *Escherichia coli*  *Escherichia coli* | 2  17 | 0  60 | No  NA | 4 | 0 | Yes |
|  |  |  |  |  |  |  |
| *Pseudomonas aeruginosa** | 4 | 40 | No | 0 | 0 | No |
| *Pseudomonas putida** | 27 | 1910 | No | 0 | 0 | No |
| *Enterobacter cloacae* | 28 | 1220 | Yes | 0 | 0 | No |
| Days 29-90 | | | | | | |
| *Escherichia coli* | 32 | 30 | NA | 4 | 0 | Yes |
| *MSSA* | 29 | 3156 | No | 3 | 0 | No |
| *Pantoea agglomerans,*  *Escherichia coli*  *Enterococcus gallinarum* | 73 | 2920 | No | 1 | 0 | No |

+Each row represents a single patient and each patient could contribute ≥1 infection; ^ANC or ALC from lab obtained closest to time of onset of infectious episode; *Organisms susceptible to levofloxacin. Abbreviations: CART; chimeric antigen receptor T cell therapy; ANC, absolute neutrophil count; ICU, intensive care unit; CRS, cytokine release syndrome; NTX, neurotoxicity; carHLH: CAR-associated hemophagocytic lymphohistiocytosis; NA, not applicable (patient in ICU at time of infection)

**Supplemental Table 3. Viral infections Pre and Post CAR T cell Therapy**

| **Infection*** | **Pre CART**  **(Day -30 to 0)**  **N=39** | **Early Post CART**  **(Day 1 to 28)**  **N=39** | **Late Post CART**  **(Day 29 to 90)**  **N=33** |
| --- | --- | --- | --- |
| Respiratory | 3 patients  HRV/ADV/FLU A (1)  FLU B 9 (1)  HRV (1) | 2 patients  HRV (1)  ADV (1) | 1 patient  PIV2(1) |
| Systemic | 7 patients  CMV (1)  EBV (1)  CMV/EBV (1)  CMV/HHV6 (1)  CMV/EBV/HHV6(1) | 4 patients  ADV (1)  EBV (2)  HHV6(1) | 1 patient  Parvovirus B-19(1) |
| Gastrointestinal | 1 patient  ADV (1) | 2 patients  ADV (2) | 4 patients  ADV (3)  Norovirus (1) |
| Other | 2 patients  BKV (urine) (1)  HSV1 (oral mucosa) (1) | 1 patient  BKV (urine/blood) (1) | - |
| **Total Patients (n; %)** | **12 (30.7 %)** | **8(20.5 %)** | **6(18.2%)** |

*Each patient could include 1 or more virus per episode. Abbreviations: CART; chimeric antigen receptor T cell therapy; ADV: adenovirus, BKV: BK virus, CMV: Cytomegalovirus, EBV: Epstein-Barr Virus, Flu A/B: Influenza virus A and B, HSV1: Human herpesvirus 1, HHV6: Human herpesvirus 6, HRV: human rhinovirus, PIV 2: Parainfluenza virus 2

**Supplemental Table 4. Infections in patients receiving ≥ 2 CAR T cell infusions**

| **Patient** | **Infusion #** | **Episodes** | **Infection** | **Days post CART** |
| --- | --- | --- | --- | --- |
| 1 | 2 | 2 | CDAD | 36 |
|  |  |  | *Pseudomonas mendocina* (BSI) | 74 |
| 1 | 4 | 2 | Parainfluenza virus 3 | 58 |
|  |  |  | Human rhinovirus/enterovirus | 76 |
| 2 | 2 | 1 | CDAD | 83 |
| 3 | 2 | 3 | Cytomegalovirus | 62 |
|  |  |  | HHv6 | 71 |
|  |  |  | CDAD |  |
|  |  |  | VGS (BSI) |  |
|  |  |  | BKV (urine/blood) | 76 |
| 4 | 2 | 2 | *VGS* and *K. pneumoniae* (BSI) | 66 |
|  |  |  | HHv6 | 82 |

Abbreviations: CART; chimeric antigen receptor T cell therapy; BSI, Blood stream infection; CDAD, C. difficile associated diarrhea; HHV6, Human herpesvirus 6; BKV, BK Virus; VGS, Viridans Group Streptococci.
